# Association Between Spinal Manipulative Therapy for Low Back Pain With or Without Sciatica and Opioid Use Disorder: A Retrospective Cohort Study

**DOI:** 10.1002/hsr2.71267

**Published:** 2025-09-19

**Authors:** Robert J. Trager, Zachary A. Cupler, Jordan A. Gliedt, Ryan A. Fischer, Roshini Srinivasan, Hannah Thorfinnson

**Affiliations:** ^1^ Connor Whole Health University Hospitals Cleveland Medical Center Cleveland Ohio USA; ^2^ Department of Family Medicine and Community Health, School of Medicine Case Western Reserve University Cleveland Ohio USA; ^3^ Department of Biostatistics and Bioinformatics Clinical Research Training Program Duke University School of Medicine Durham North Carolina USA; ^4^ Physical Medicine & Rehabilitative Services Butler VA Health Care System Butler Pennsylvania USA; ^5^ Department of Neurosurgery Medical College of Wisconsin Milwaukee Wisconsin USA; ^6^ Section of General Internal Medicine, Department of Medicine Boston University Chobanian & Avedisian School of Medicine and Boston Medical Center Boston Massachusetts USA; ^7^ Duke University Department of Medicine Duke University Hospital Durham North Carolina USA; ^8^ Butler VA Medical Center Butler VA Health Care System Butler Pennsylvania USA

**Keywords:** adverse drug event, analgesics, chiropractic, opioid‐related disorders, opioids, spinal manipulation

## Abstract

**Background and Aims:**

Previous research suggests patients receiving spinal manipulative therapy (SMT) for low back pain (LBP) are less likely to be prescribed opioids. However, the clinical implications of this finding are unclear. We tested the hypothesis that opioid‐naïve adults receiving SMT for LBP are less likely to develop opioid use disorder (OUD) compared to matched controls prescribed ibuprofen over 2 years follow‐up.

**Methods:**

We queried a United States data resource (TriNetX) for patients age ≥ 18 years with a new episode of LBP with/without sciatica from 2015 to 2023 (allowing for up to 2 years of follow‐up to 2025), excluding those with serious pathology, OUD, and opioid prescription. We divided patients into cohorts: (1) SMT and (2) ambulatory ibuprofen prescription, using propensity matching for OUD risk factors. Our primary outcome was the risk ratio (RR) of OUD. We secondarily explored the RR for long‐term opioid use, and opioid prescription RR and mean count. Primary analyses conducted in TriNetX and R used logistic regression for matching, standardized mean difference to assess between‐cohort balance (threshold of ≤ 0.1), and contingency tables for RRs, using a significance threshold of *p* < 0.05.

**Results:**

24,993 patients remained per cohort following matching. Comparing the SMT cohort to ibuprofen cohort, there was a significantly lower incidence and risk of OUD [95% CI] (0.24% vs. 1.51%; RR = 0.20 [0.15, 0.28]; *p* < 0.001), long‐term opioid use (0.42% vs. 1.85%; RR = 0.23 [0.18, 0.28]; *p* < 0.001), and opioid prescription (30.96% vs. 45.00%; RR = 0.69 [0.67, 0.71; *p* < 0.001]). SMT recipients also received fewer opioid prescriptions [standard deviation] (1.0 [3.3] vs. 2.1 [5.7]; *p* < 0.001).

**Conclusion:**

Adults receiving SMT for new LBP had a lower risk of OUD compared to matched controls prescribed ibuprofen. These findings corroborate guidelines recommending first‐line SMT for LBP. The role of SMT in mitigating opioid‐related harms requires further investigation.

## 1 Background

Low back pain (LBP) is a highly prevalent condition and represents the leading cause of years lived with disability worldwide per the 2019 Global Burden of Disease study [1]. In the United States (US), LBP is a leading reason for seeking healthcare services and ranks among the costliest conditions for healthcare expenditures [2, 3]. LBP is managed through a variety of pharmacological and nonpharmacological approaches. However, care utilization and downstream health impacts may depend on the first provider seen and/or initial treatments received [4, 5].

Opioid use disorder (OUD) is a complex disease characterized by chronic use of opioids leading to substantial distress or impairment in daily life [6]. The Diagnostic and Statistical Manual of Mental Disorders (DSM‐5) delineates criteria for OUD based on behavioural, psychological, and physiological markers [7]. In research, OUD is often broadly defined to include related adverse events including opioid overdose and medication for OUD [8, 9, 10, 11]. Unless otherwise noted, this study adopts the broader definition.

Opioids are narcotic analgesic medications commonly prescribed for LBP, despite their known risks (e.g., constipation, addiction, and overdose) and limited evidence supporting their efficacy. In a United States (US) study examining data from 2009 to 2017, opioids were prescribed to one in five patients with new‐onset LBP [12]. A network meta‐analysis in 2022 reported that opioids were not superior to exercise or heat therapy for acute or subacute LBP and led to greater adverse events [13]. The prevalence of OUD among US adults in 2019 was 2.0% to 2.8% [14], and slightly more common among adults with LBP at approximately 3% [15, 16]. Given the risks of opioid prescription, including OUD, the US Centers for Disease Control and Veterans Health Administration encourage the use of nonpharmacologic treatments for acute LBP [17, 18].

Spinal manipulative therapy (SMT) is a common treatment for LBP often used by chiropractors in the US [19]. SMT is also used by other disciplines, including physical therapists, doctors of osteopathy, and internationally by practitioners of traditional East Asian medicine [19]. Several studies have found that those visiting a chiropractor and/or receiving SMT are less likely to be prescribed opioids [20, 21, 22, 23, 24, 25, 26, 27, 28]. While higher opioid prescription volume is a risk factor for OUD [29], it remains unclear whether providers' reduced prescribing behavior translates to a lower risk of OUD for patients. The present study focuses on chiropractor‐delivered SMT, as it is the most common form of SMT in the US and can be reliably identified in large US health records databases using specific procedural terminology codes.

Research on opioid‐related outcomes associated with chiropractic and/or SMT is limited. One recent study identified that individuals receiving SMT for sciatica from a chiropractor were less likely to experience an opioid‐related adverse event, such as overdose or poisoning, compared to matched controls receiving usual medical care [28]. Another study found fewer adverse drug events among SMT recipients, although these included opioid‐ and non‐opioid‐related events, limiting conclusions to OUD [30]. Finally, in older adults with chronic LBP, SMT was associated with a lower incidence of “opioid dependence or abuse” compared to opioid therapy over 12 months (i.e., 0.3% vs. 14.3%) [31]. However, a comparison with opioid therapy may not translate to real‐world care, as SMT is a first‐line intervention for nonserious LBP, while opioids are often reserved for more severe or complex LBP and carry a heightened OUD risk [17, 29]. Examination of OUD as an outcome following SMT has yet to be conducted. The present study is the first to directly assess OUD incidence as the primary outcome of interest in SMT recipients versus an active comparator.

While SMT has been associated with a reduced likelihood of opioid prescription, and reduced rate of OUD compared to opioid therapies, its association with OUD risk remains unclear compared to other first‐line LBP treatments. The aim of this study was to investigate the association between SMT and the risk of developing OUD in opioid‐naïve adults with a new episode of LBP with or without sciatica, compared to propensity‐matched controls prescribed ibuprofen, over a 2‐year follow‐up. Specifically, we hypothesized that adults receiving SMT would have a reduced likelihood of developing OUD compared to those prescribed ibuprofen.

## 2 Materials and Methods

### 2.1 Study Design

This retrospective cohort study included patients meeting eligibility criteria from 2015 to 2023, with a query date of February 14, 2025, allowing for 2 years of follow‐up, adhering to a registered protocol [32]. A visual representation of the study design is available (Supplemental File 1, Figure S1).

We derived data from a US resource (TriNetX LLC.; Cambridge, MA, US), which includes 141 million patients across 104 large healthcare organisations. TriNetX complies with the Health Insurance Portability and Accountability Act [33] as data from its online platform are aggregated and deidentified. Data chiefly stem from data electronic health records, being routinely collected during healthcare encounters. TriNetX allows queries using nomenclatures including International Classification of Diseases, 10th Revision (ICD‐10), and Current Procedural Terminology (CPT), and includes demographics, diagnoses, procedures, and test results. Mortality data are pooled from diagnosis codes, health records, claims, Social Security Administration Master Death File, and/or private obituaries, and are refreshed/updated approximately every two to 4 weeks. TriNetX regularly conducts quality assurance measures. One study estimated that the completeness of prescribed medication data in TriNetX was least 87% [34].

The University Hospitals Institutional Review Board (IRB; Cleveland, OH, US) considers studies using deidentified data from the online TriNetX platform (TriNetX Inc.; Cambridge, MA, US) to represent ‘Not Human Subjects Research’ thereby exempting this study from IRB review and waiving the requirement for consent (IRB number: STUDY20250510).

### 2.2 Participants

We included adults at least 18 years old, receiving care for a new LBP episode with or without sciatica (ICD‐10: M54.4 or M54.5). To improve data completeness, we required patients to have a healthcare visit between 1 day and 2 years before the index date of inclusion. To minimize loss to follow‐up, we required patients to have at least one additional healthcare visit or have a recorded status of ‘deceased’ during the 2‐year follow‐up.

We used an active comparator to help ensure similar complexity and engagement in LBP care‐seeking between cohorts. We selected ibuprofen for this purpose because it is a nonsteroidal anti‐inflammatory drug (NSAID), a commonly prescribed medication class for new LBP episodes [35, 36]. Similar to SMT, ibuprofen is inexpensive, widely covered by insurance for LBP [37, 38], and used in ambulatory settings [35, 36]. Both SMT and ibuprofen are relevant first‐line LBP interventions recommended by practice guidelines, supporting their comparability [39, 40, 41]. Recipients of SMT from chiropractors have a similar likelihood of being prescribed NSAIDs compared to nonrecipients, suggesting that patient preferences for or against NSAIDs would not meaningfully influence our findings [22, 42]. Finally, there is no strong evidence that ibuprofen increases the risk of OUD, our primary outcome [29].

We divided patients into two cohorts dependent on the treatment received on the index date of a new LBP episode with or without sciatica: (1) SMT; those receiving any CPT code delivered by a chiropractor for this procedure (98940, 98941, 98942); and (2) ibuprofen; those prescribed ibuprofen (RxNorm: 5640) at an ambulatory visit and not receiving SMT on that date.

To create opioid‐naïve cohorts, we excluded patients with any concurrent or prior opioid prescription in the preceding year. We excluded those with serious pathology (e.g., cauda equina syndrome, spinal infection, bleed, fracture, and cancer), and specific conditions causing spinal pain (e.g., multiple sclerosis, myelopathy, plexopathy, and spinal deformity). We excluded pregnant people and those receiving palliative or hospice care [17]. We excluded those with previously‐diagnosed OUD and those at much higher risk of OUD: those with an opioid‐related adverse event in the preceding year [17], a diagnosis of cocaine or stimulant use disorder, positive urine test for fentanyl, methamphetamine, or cocaine, or prescription of fentanyl, sufentanil, or hydromorphone (i.e., highly potent opioids) [43, 44], those prescribed medication for OUD (i.e., methadone and buprenorphine) [45], and those with recent anesthesia or spinal surgery [46]. We also excluded those prescribed naloxone in the preceding year who are likely at higher risk of opioid exposure. We excluded patients from the ibuprofen cohort who received SMT on the index date of diagnosis. To create new episodes of LBP, we excluded patients with any LBP‐related care in the preceding 3 months using a range of LBP diagnoses. Exclusions are summarized in Supplemental File 1, Table S1.

### 2.3 Confounding Variables

We propensity matched cohorts to minimize confounding, matching covariates present within a year preceding and including the date of inclusion associated with OUD (Supplemental File 1, Table S2). To ensure that the likelihood of taking medications was similar across cohorts, we matched on receipt of any prescription [47]. Given the potential for temporal changes in prescribing practices and associated OUD risk [48], we matched cohorts based on age at the index date as well as their age at the time of our query. This helped ensure that the years of observation remained similar between cohorts.

### 2.4 Primary Outcome

We identified instances of OUD using a composite of diagnoses, medications, and supply codes, which include opioid‐related disorders (ICD‐10: F11), opioid overdoses, and OUD treatment interventions, including medication for opioid dependence and services provided within outpatient, inpatient, and residential opioid treatment programs [8, 9, 10, 11, 49, 50] (Supplemental File 1, Table S3). This broad strategy is needed given the variability in coding for OUD [9, 51], is supported by similar methods from the US Department of Health and Human Services [8] and other publications [9, 10, 11, 49, 50], and aims to increase the ascertainment of OUD. This outcome does not include generic codes related to alcohol or other substance use disorders, codes focused on non‐prescription drug use or assault, or naloxone prescriptions, which are often given prophylactically in take‐home programs and may not accurately reflect an active OUD [52]. The ascertainment period began the day after the index date and extended through a 2‐year follow‐up. A 2‐year window was used accounting for time to develop OUD [53], and potential lags in documenting OUD in the health record. As a sensitivity analysis, we narrowed the definition of OUD relying solely on the ICD‐10 code for opioid‐related disorders (i.e., F11). This code has a high predictive value of 96%, yet a low sensitivity of 43% [54]. Accordingly, it may underestimate the incidence of OUD yet provide a stricter comparison while minimizing misclassification.

### 2.5 Secondary Outcomes

As a secondary outcome, we compared the likelihood and mean count of new opioid prescriptions between cohorts. This outcome was used to provide additional insights into our primary outcome, considering opioid prescription volume is an OUD risk factor [29]. Additionally, we compared the likelihood of long‐term opioid therapy between cohorts (ICD‐10: Z79.891). While this diagnosis code does not appear in previous research‐based definitions of OUD [8, 9, 10, 11, 49, 50], it is related and aligns with DSM‐5 criteria [51, 55]. Long‐term opioid use is generally defined as use for at least 90 days, though real‐world use of the ICD code may use variable thresholds [56].

### 2.6 Statistical Methods

We used the features of the TriNetX online analytics suite to compare baseline characteristics before and after propensity score matching using standardized mean difference (SMD), with a threshold of ≤ 0.1 to assess between‐cohort balance. Propensity scores were calculated using logistic regression implemented in Python (scikit‐learn, Python Software Foundation, DE, USA). The model computed the log odds of being prescribed ibuprofen as a function of the covariate matrix. Each patient received a propensity score, ranging from 0 (indicating the lowest likelihood of being in the ibuprofen cohort) to 1 (highest likelihood). To balance cohorts, 1:1 greedy nearest neighbor matching was applied with a tolerance of 0.1 pooled standard deviations of the logit of the propensity score. We did not impute missing data.

We used R (build 402, Vienna, AT [57]) to calculate risk ratios (RRs) for OUD, opioid prescription, and long‐term opioid use by dividing incidence in the SMT cohort by the ibuprofen cohort, providing 95% confidence intervals (CI) for each. We calculated the mean and standard deviation (SD) for opioid prescription, which was compared using a T‐test with a significance threshold of *p* < 0.05. We calculated risk difference (RD) and its 95% CI using the Miettinen and Nurminen method, via the DescTools package [58], and calculated *p*‐values for RRs and risk difference using the chi‐square test with a significance threshold of 0.05. Contingency tables were used to calculate RRs and *p*‐values for OUD and other opioid‐related outcomes. We reported OUD incidence per 100,000 patient‐years and CI for incidence proportions per cohort, and used ggplot2 [59] to plot total and cumulative incidences along with 95% CI.

To assess data quality and completeness, we reported cohorts' duration of follow‐up (i.e., mean [compared via SMD], median, and interquartile range), proportion of unknown variables, and plotted propensity score density and covariate balance using ggplot2 [59]. Patients were censored after the last clinical fact in their record and not censored for mortality. We explored RRs for negative control outcomes unrelated to SMT [60] (Supplemental File 1, Table S4), with the aim of at least half of negative control RRs falling within a pre‐defined range suggestive of between‐cohort balance (0.73 ≥ RR ≤ 1.38) [60, 61]. Forest plots for negative control outcomes were generated using R and ggplot2 to visualize RRs and assess between‐cohort balance.

### 2.7 Study Size

We calculated a total required sample size of 17,550 based on previous prevalence estimates of OUD among those with LBP [15, 16]. We used G*Power (Kiel University, DE), Z‐tests for determining a difference between two independent proportions (0.03 vs. 0.04), using an allocation ratio of one, two‐tailed α error of 0.05, and power of 0.95.

## 3 Results

### 3.1 Patients

Before matching, the SMT cohort had 25,274 patients, while the ibuprofen cohort had 597,406. After matching, both cohorts had 24,993 patients. Before matching, the ibuprofen cohort had a meaningfully larger proportion of patients who identified as Black or African American, patients prescribed sedatives/hypnotics, patients previously diagnosed with substance use disorders other than opioid‐related disorders, and meaningfully smaller proportion of patients who identified as White, among other differences (SMD > 0.1). Following matching, all characteristics were balanced (SMD < 0.1). Baseline characteristics are summarized in Table 1.

**Table 1:** Baseline characteristics before and after propensity score matching.

Variable *n* (%) or mean (SD)	Before matching			After matching		
	SMT	Ibuprofen	SMD	SMT	Ibuprofen	SMD
Patients *n*	25274	597406	NA	24993	24993	NA
Age at index	51.7 (17.2)	49.6 (16.3)	0.127	51.6 (17.2)	51.5 (16.8)	0.009
Age at query	57.5 (17.3)	55.0 (16.2)	0.152	57.4 (17.3)	57.3 (16.8)	0.008
Female	14743 (58%)	400568 (67%)	0.181	14649 (59%)	14728 (59%)	0.006
Male	10530 (42%)	196787 (33%)	0.181	10343 (41%)	10259 (41%)	0.007
Family history of substance abuse and dependence	0 (0%)	43 (0%)	0.012	0 (0%)	0 (0%)	0.000
Unknown sex	≤ 10 (0%)	51 (0%)	0.020	≤ 10 (0%)	≤ 10 (0%)	0.000
Mood disorders	3837 (15%)	121665 (20%)	0.136	3831 (15%)	3825 (15%)	0.001
Black or African American	1179 (5%)	150352 (25%)	0.601	1179 (5%)	1183 (5%)	0.001
Medications (Any)	21896 (87%)	570141 (95%)	0.312	21889 (88%)	21898 (88%)	0.001
Alcohol related disorders	361 (1%)	19155 (3%)	0.118	361 (1%)	369 (1%)	0.003
Sedatives/hypnotics	3480 (14%)	168745 (28%)	0.361	3480 (14%)	3440 (14%)	0.005
Personal history of psychological trauma	11 (0%)	782 (0%)	0.030	11 (0%)	14 (0%)	0.005
Alcohol deterrents	124 (0%)	3303 (1%)	0.009	124 (0%)	114 (0%)	0.006
Suicide attempt	≤ 10 (0%)	663 (0%)	0.026	≤ 10 (0%)	14 (0%)	0.007
Personal history of self‐harm	13 (0%)	2095 (0%)	0.067	13 (0%)	18 (0%)	0.008
Spinal stenosis, cervical	153 (1%)	8571 (1%)	0.083	153 (1%)	137 (1%)	0.008
Chronic pain	5131 (20%)	158348 (27%)	0.147	5125 (21%)	5216 (21%)	0.009
Nerve root and plexus disorders	214 (1%)	1930 (0%)	0.069	200 (1%)	180 (1%)	0.009
Critical care services	150 (1%)	10630 (2%)	0.110	149 (1%)	131 (1%)	0.010
Hispanic or Latino	532 (2%)	63306 (11%)	0.354	532 (2%)	569 (2%)	0.010
Suicidal ideations	61 (0%)	7474 (1%)	0.118	61 (0%)	76 (0%)	0.011
Adverse socioeconomic & psychosocial factors	140 (1%)	13775 (2%)	0.148	140 (1%)	164 (1%)	0.012
Anxiety disorders	5034 (20%)	132170 (22%)	0.054	5012 (20%)	4888 (20%)	0.012
Lumbago with sciatica	2449 (10%)	86376 (14%)	0.147	2433 (10%)	2328 (9%)	0.014
Tobacco use	129 (1%)	22866 (4%)	0.229	129 (1%)	157 (1%)	0.015
Substance use disorder (other than opioid‐related disorders)	1587 (6%)	97766 (16%)	0.322	1586 (6%)	1474 (6%)	0.019
Other race	451 (2%)	22798 (4%)	0.123	451 (2%)	390 (2%)	0.019
Chronic viral hepatitis C	24 (0%)	4841 (1%)	0.107	24 (0%)	42 (0%)	0.020
Nicotine dependence	1267 (5%)	79166 (13%)	0.289	1266 (5%)	1148 (5%)	0.022
Vitamin D deficiency	1568 (6%)	64644 (11%)	0.166	1566 (6%)	1425 (6%)	0.024
Surgery	16892 (67%)	334078 (56%)	0.226	16692 (67%)	16979 (68%)	0.024
Radiculopathy	3756 (15%)	59291 (10%)	0.150	3695 (15%)	3479 (14%)	0.025
Osteoarthritis	1634 (6%)	55057 (9%)	0.102	1623 (6%)	1468 (6%)	0.026
Inflammatory polyarthropathies	1144 (5%)	33255 (6%)	0.048	1131 (5%)	987 (4%)	0.029
Sciatica	1051 (4%)	31693 (5%)	0.054	1038 (4%)	868 (3%)	0.036
White	19535 (77%)	364500 (61%)	0.358	19494 (78%)	19882 (80%)	0.038
Unknown race	3724 (15%)	33528 (6%)	0.305	3484 (14%)	3155 (13%)	0.039
Not Hispanic or Latino	21345 (84%)	472048 (79%)	0.141	21283 (85%)	21866 (87%)	0.068
Unknown ethnicity	3397 (13%)	62052 (10%)	0.094	3178 (13%)	2558 (10%)	0.078

*Note:* After patients, age, and sex, values are shown in order of ascending SMD values after matching.

Abbreviations: NA, not applicable; SMD, standardized mean difference; SMT, spinal manipulative therapy.

### 3.2 Opioid Use Disorder

**Figure 1:**
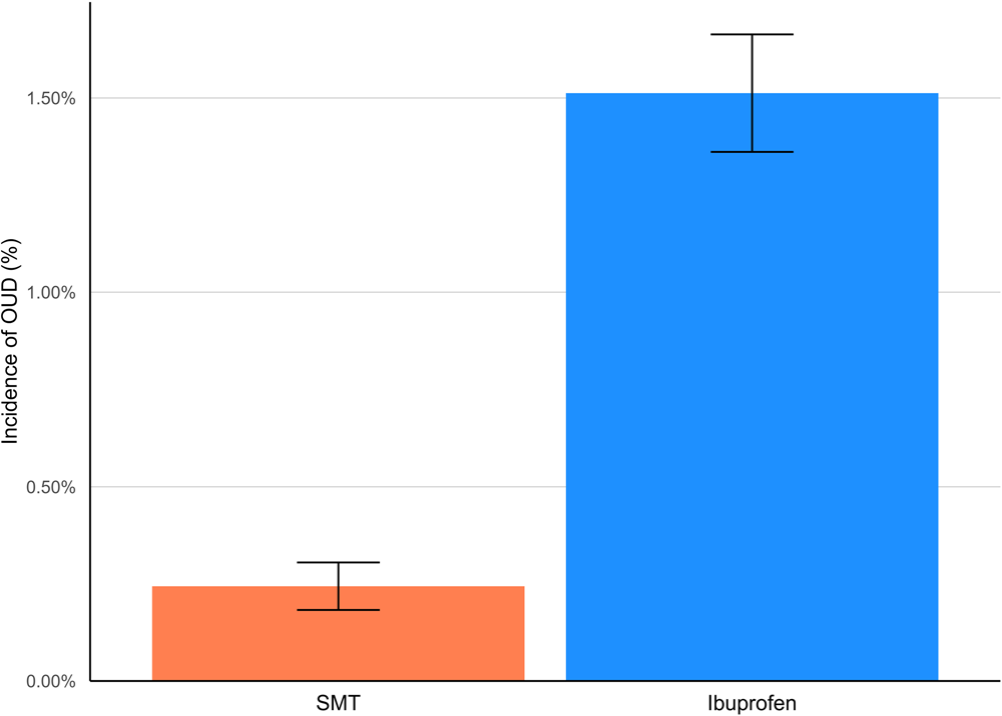
Incidences of opioid use disorder (OUD) after propensity matching. Incidence in the spinal manipulative therapy (SMT) cohort (orange) is lower than that of the ibuprofen cohort (blue). The 95% confidence intervals do not overlap, suggesting a meaningful between‐cohort difference.

**Figure 2:**
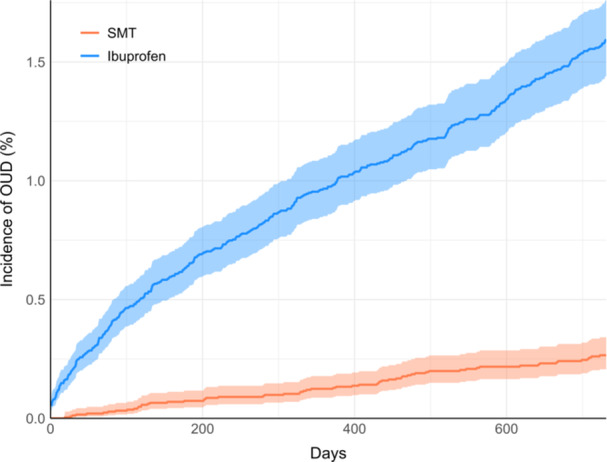
Cumulative incidences of opioid use disorder (OUD) per cohort. The incidence for the spinal manipulative therapy (SMT) cohort is shown in orange, while that of the ibuprofen cohort is shown in blue, over the 730‐day (two‐year) follow‐up. Shaded regions indicate 95% confidence intervals.

After matching, the SMT cohort had a significantly lower incidence and risk of OUD over follow‐up compared to the ibuprofen cohort [95% CI] (0.24% vs. 1.51%; RR = 0.20 [0.15, 0.28]; *p* < 0.001), yielding a risk difference of −1.27% (95% CI: −1.44%, −1.11%; *p* < 0.001). This translated to an incidence of 122 versus 756 OUD cases per 100,000 person‐years, respectively, per cohort. Similarly, our sensitivity analysis revealed a significantly lower incidence and risk of opioid‐related disorders [95% CI] (0.17% vs. 0.85%; RR = 0.20 [0.15, 0.28; *p* < 0.001]), long‐term opioid use (0.42% vs. 1.85%; RR = 0.23 [0.18, 0.28]; *p* < 0.001), and opioid prescription (30.96% vs. 45.00%; RR = 0.69 [0.67, 0.71; *p* < 0.001]) in the SMT cohort compared to the ibuprofen cohort. The SMT cohort also received fewer mean opioid prescriptions per patient over follow‐up [SD] (1.0 [3.3] vs. 2.1 [5.7]; *p* < 0.001). Quantitative results for each outcome are shown in Table 2, while total and cumulative incidences for OUD are shown in Figures 1 and 2, respectively.

**Table 2:** Key outcomes.

	Before matching		After matching	
	SMT	Ibuprofen	SMT	Ibuprofen
**Number of patients**	25274	597406	24993	24993
**Opioid use disorder** ^**a**^				
*n*	61	13394	61	378
% (95% CI)	0.24% (0.18%, 0.30%)	2.24% (2.20%, 2.28%)	0.24% (0.18%, 0.31%)	1.51% (1.36%, 1.66%)
RR (95% CI)*	0.11 (0.08, 0.14; *p* < 0.001)	NA	0.16 (0.12, 0.21; *p* < 0.001)	NA
RD (95% CI)	−2.00% (−2.07%, −1.92%; *p* < 0.001)	NA	−1.27% (−1.44%, −1.11%; *p* < 0.001)	NA
**Opioid‐related disorders** ^b^				
*n*	43	8544	43	212
% (95% CI)	0.17% (0.12%, 0.22%)	1.43% (1.40%, 1.46%)	0.17% (0.12%, 0.22%)	0.85% (0.73%, 0.96%)
RR (95% CI)	0.12 (0.09, 0.16; *p* < 0.001)	NA	0.20 (0.15, 0.28; *p* < 0.001)	NA
RD (95% CI)	−1.26% (−1.31%, −1.19%; *p* < 0.001)	NA	−0.68% (−0.81%, −0.56%; *p* < 0.001)	NA
**Long‐term opioid use** ^c^				
*n*	106	13957	106	463
% (95% CI)	0.42% (0.34%, 0.50%)	2.34% (2.30%, 2.37%)	0.42% (0.34%, 0.50%)	1.85% (1.69%, 2.02%)
RR (95% CI)	0.18 (0.15, 0.22; *p* < 0.001)	NA	0.23 (0.18, 0.28; *p* < 0.001)	NA
RD (95% CI)	−1.92% (−2.00%, −1.82%; *p* < 0.001)	NA	−1.43% (−1.62%, −1.25%; *p* < 0.001)	NA
**Opioid prescription** ^c^				
*n*	7780	288007	7739	11247
% (95% CI)	30.78% (30.21%, 31.35%)	48.21% (48.08%, 48.34%)	30.96% (30.39%, 31.54%)	45.00% (44.38%, 45.62%)
RR (95% CI)	0.64 (0.62, 0.65; *p* < 0.001)	NA	0.69 (0.67, 0.71; *p* < 0.001)	NA
RD (95% CI)	−17.43% (−18.01%, −16.84%; *p* < 0.001)	NA	−14.04% (−14.88%, −13.19%; *p* < 0.001)	NA
Mean count (SD)	1.0 (3.2) *p* < 0.001	2.5 (7.5)	1.0 (3.3) *p* < 0.001	2.1 (5.7)

*Note:* Primary outcome (^a^), sensitivity analysis (^b^), secondary outcome (^c^).

Abbreviations: RD, risk difference; RR, risk ratio; SD, standard deviation; SMT, spinal manipulative therapy; 95% CI, 95% confidence intervals.

### 3.3 Data Quality

**Figure 3:**
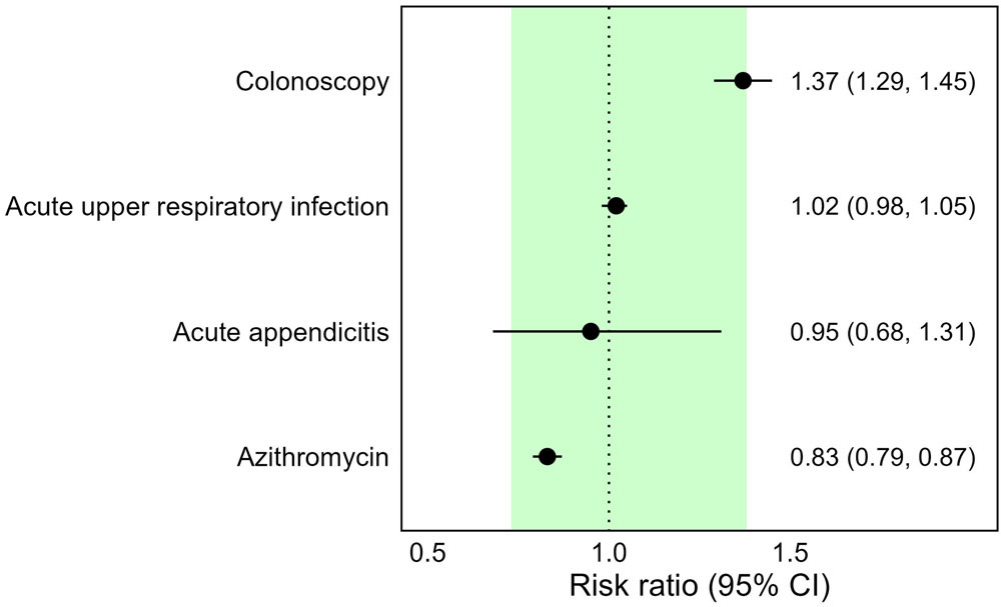
Negative control outcomes after matching. Point estimates (dots) and 95% confidence intervals (CI; error bars) are shown for risk ratios for each outcome. The green area (point estimates of 0.73 to 1.38) represents the predefined bounds for negative control outcome balance, while the vertical dashed line indicates the null value of 1.0.

After matching, propensity score densities overlapped and all SMD values were less than 0.1, including those for unknown sex, race, and ethnicity, suggesting adequate matching (Supplemental File 1, Figures S2 and S3, respectively). Negative control outcome point estimates fell within the predefined range approximating the null, further supporting between‐cohort balance (Figure 3). The mean follow‐up duration was similar between cohorts [SD] (SMT: 671 days [155]; ibuprofen 686 days [140]; SMD = 0.100), while the median follow‐up was 730 days (interquartile range of 0) for both cohorts. The proportion of patients having at least 2 years’ follow‐up data exceeded 80% in both cohorts (SMT: 81.1%; ibuprofen: 86.6%; Supplemental File 1, Figure S4). The total observation years was 45,901 (SMT) and 46,914 (ibuprofen). Together, these findings suggest adequate covariate balance and minimal impact of loss to follow‐up.

## 4 Discussion

The present study findings support the hypothesis that adults receiving SMT for LBP with or without sciatica have a lower risk of developing OUD compared to those receiving ibuprofen. Additional findings corroborate this primary outcome considering SMT recipients were also less likely to be diagnosed with opioid‐related disorders, have long‐term opioid use, or be prescribed an opioid.

The incidence of OUD and other outcomes in the present study should be compared with external studies cautiously, given differences in study populations and methods. Our observed value in the ibuprofen cohort was 1.51% over 2‐years’ follow‐up, which is less than prior estimates of approximately 3% among LBP sufferers [15, 16]. However, the latter estimates derived from older adult populations at least an average of 10 years older than our study population [15, 16]. Additionally, the present incidence values may have been influenced by including more recent data compared to the previous studies. Illustratively, our data range including follow‐up was 2015 to 2025, compared to previous studies which included data from 2016 to 2018 or 2019 [15, 16]. Recent guidelines from the American College of Physicians (2018) and Center for Disease Control (2022) have encouraged nonpharmacologic interventions rather than opioids for LBP [17, 39], thereby potentially reducing our observed OUD incidence. Our study was also unique in that we excluded patients with opioid prescriptions in the preceding year, as well as those with previous OUD, thereby likely reducing the observed incidence of OUD across cohorts.

Our observed reduced risk of long‐term opioid use among SMT recipients corroborates the findings of a prior study by Azad, et al., who estimated a risk of long‐term opioid use of 2.03% (95% CI: 1.95%, 2.1%) among patients receiving primary care for LBP with or without lower extremity pain, and a lower risk among chiropractic care recipients of 0.98% (95% CI 0.88, 1.08%) [23]. While our study found slightly lower incidence estimates overall, both our study and the prior studies observed associations favoring a lower risk of long‐term opioid use among chiropractic care recipients. Additionally, lower overall incidences of long‐term opioid use in our study may be partly explained by our exclusion of patients with prior OUD. Long‐term opioid use should be interpreted with caution considering it is a risk factor for OUD and does not, by itself, equate to OUD [56].

Our observed reduction in risk of receiving an opioid prescription may account for the reduction in risk of OUD among SMT recipients. The reduction in opioid prescriptions among SMT recipients corroborates previous studies that observed similar findings [20, 21, 22, 23, 24, 25, 26, 27, 28]. While the mechanisms involved in reduced opioid prescriptions remain unclear, we propose two main hypotheses. First, SMT and associated patient education may provide adequate LBP relief [39, 40, 41] such that patients are not prompted to seek pharmaceutical forms of analgesia. Second, considering that chiropractors are portal‐of‐entry clinicians and do not prescribe opioids, the results may be a function of the initial care pathway received for this disorder, with non‐chiropractic clinicians being more likely to render an opioid prescription as opposed to other nonpharmacological interventions or non‐opioid medication alternatives [23]. Our matching strategy, which accounted for previous medication use, suggests that a general preference towards or against medication use in either cohort would not account for our findings.

The present study, therefore, builds upon previous work to examine questions of clinical relevance and opioid‐related harms as it pertains to SMT for patients with spinal pain. A prior, similar study which involved our team identified a reduced risk of opioid‐related adverse events such as overdoses and poisonings among adults receiving SMT for sciatica compared to those receiving usual care [28]. The present study extends that work to examine a broader outcome of OUD, encompassing not only adverse events, but markers of use disorder and medication management for OUD. Additionally, the present study included a broader definition of LBP not limited exclusively to sciatica, which has greater generalizability. Although modest, the absolute difference in OUD risk may have meaningful public health implications: OUD is a costly, chronic, often relapsing disorder that often requires long‐term medical, social, and behavioural support [62]. OUD is also associated with economic and societal costs, lost work productivity, increased healthcare use, and harms to family cohesion [16, 62]. Thus, SMT for LBP may have broader health impacts, which require additional investigation.

The present findings support existing clinical guidelines that recommend SMT as a nonpharmacological intervention for LBP with or without sciatica in adults [39, 40, 41]. While the present findings are not entirely conclusive, they corroborate previous work, which collectively suggests that access to SMT as a first‐line treatment for LBP could contribute to opioid‐sparing efforts and reductions in opioid‐related sequelae [20, 21, 22, 23, 24, 25, 26, 27, 28]. The present findings may further corroborate established guideline recommendations to influence care. Illustratively, to prioritize non‐pharmacologic care in LBP management and potentially mitigate OUD risk in opioid‐naïve patients with new LBP, primary care providers and pain management specialists could consider early care pathways to chiropractors for SMT. Ultimately, clinical decisions to enter a care pathway for SMT versus other interventions should be guided and informed by clinical appropriateness, patient preferences, cost‐effectiveness, and availability or accessibility to services.

Our findings, suggesting a reduced risk of OUD associated with SMT for LBP, may have implications for health policy. Payers, including Medicare and Medicaid, such as in this current study, could consider real‐world data to support expanding coverage and reimbursement for nonpharmacologic treatments like SMT, which often faces stricter condition requirements, visit limits, and/or higher out‐of‐pocket costs compared to other conservative care options [38]. These results may inform value‐based care models and justify improved access and payment structures that prioritize opioid‐sparing interventions. Such changes could ultimately mitigate OUD risk and downstream care cascades in LBP management.

Given the rarity and duration of follow‐up needed to examine OUD as an outcome, this topic may be challenging to examine via randomized controlled trials. However, future observational studies could expand upon our findings by examining whether the observed reduction in OUD risk is unique to SMT delivered by chiropractors or extends to other forms of nonpharmacologic care, such as therapeutic exercise, spinal manipulation delivered by other health care professionals, acupuncture, or education/advice [63]. Another active control cohort such as acetaminophen could also help determine whether the findings are stable across pharmacologic comparators commonly used for LBP [36, 40, 41]. Additionally, more narrowly focused studies could help determine whether the association varies across subtypes of spinal pain (e.g., neck pain, sciatica, stenosis) or other population groups such as Veterans or older adults.

### 4.1 Strengths and Limitations

Strengths of this study included a large sample size, multidisciplinary author team representing chiropractic, pharmacy, and internal medicine, a comprehensive propensity matching strategy, and robust data quality metrics.

However, limitations should be noted. First, we are unable to conclude that potential changes in OUD risk may be caused by SMT given the observational nature of the study. Second, we are unable to validate our query or test its accuracy against manual chart review. Third, we are unable to determine measures of LBP intensity (e.g., visual analog scale) or disability (e.g., patient‐reported outcome assessments). Fourth, while medication completeness in TriNetX is suitable for real‐world research, this resource may miss a small percentage of prescriptions due to care fragmentation, for instance, if patients visited healthcare organizations not linked to the data set [34]. Fifth, we were unable to determine morphine equivalent daily dosages, severity of OUD diagnoses, or describe the number of clinical OUD features per patient. Sixth, clinicians may have appended OUD diagnoses using variable thresholds, yet we have no reason to suspect this would differ between cohorts. Seventh, there may be residual unmeasured confounding related to unreported non‐prescription substance or naloxone use, the number of unique opioid prescribers [64], LBP severity or related disability, adverse socioeconomic and psychosocial circumstances [17], patient care preferences, or adherence to clinical recommendations. Eighth, comparing ibuprofen (pharmacological) to SMT (nonpharmacological) may introduce selection bias due to confounding by indication or biases related to patients’ preferences toward either treatment mechanism [65]. Although successful indicators of propensity score matching on prior medications and a balanced negative control outcome for azithromycin suggested a lack of such bias, unmeasured between‐cohort differences in patient preferences may have remained.

Finally, these findings may not be generalizable to smaller private healthcare settings or countries outside of the US, where opioid prescribing practices or LBP management may vary. Specifically, differences in access to chiropractors (e.g., referral *vs.* direct) or availability of chiropractors and other SMT practitioners as a workforce, prescribing scope of providers, opioid prescribing policies, healthcare models (e.g., fee‐for‐service in the US *vs.* universal healthcare systems), and patient preferences for pharmacologic versus non‐pharmacologic treatments may limit generalizability internationally.

## 5 Conclusion

In this retrospective cohort study, adults receiving SMT for LBP with or without sciatica had a significantly lower risk of developing OUD over a 2‐year follow‐up compared to those prescribed ibuprofen. These findings align with prior research associating SMT with reduced opioid prescription and related harms. These results highlight the potential role of SMT as a guideline‐concordant opioid‐sparing LBP intervention. Future research should explore whether similar associations exist across other forms of nonpharmacologic care and in different patient populations [66].

## Author Contributions

**Robert J Trager:** conceptualization, methodology, investigation, software, formal analysis, resources, data curation, writing – original draft, visualization, supervision, project administration, funding acquisition, writing – review and editing. **Zachary A Cupler:** conceptualization, methodology, investigation, writing – review and editing. **Jordan A Gliedt:** conceptualization, methodology, investigation, writing – review and editing. **Ryan A Fischer:** methodology, investigation, writing – review and editing. **Roshini Srinivasan:** methodology, investigation, writing – review and editing. **Hannah Thorfinnson:** methodology, investigation, writing – review and editing.

## Ethics Statement

The University Hospitals Institutional Review Board (IRB; Cleveland, OH, US) considers studies using deidentified data from the online TriNetX platform (TriNetX Inc.; Cambridge, MA, US) to represent ‘Not Human Subjects Research’ thereby exempting this study from IRB review and waiving the requirement for consent (IRB number: STUDY20250510).

## Conflicts of Interest

Robert J. Trager acknowledges that he has received royalties as the author of two texts on the topic of sciatica. The other authors have declared no competing interests.

## Supporting information


**Figure S1:** Graphical representation of study design. The vertical gray arrow indicates the index date (cohort entry date ‐‐ day 0), representing is the when spinal manipulative therapy (SMT) or ibuprofen are received and other criteria are met. Text and boxes describe study eligibility criteria which were assessed during time windows ([#, #]) in days relative to the index date. Figure created by Robert J. Trager using a Creative Commons template from Wang et al [1]. **Figure S2:** Propensity score density graph. Density scores before (A) and after (B) matching. Orange bars represent the chiropractic spinal manipulative therapy (SMT) cohort while blue bars represent the ibuprofen control cohort. After matching, densities overlap closely (shown in gray), suggesting adequate balance of covariates. **Figure S3:** Covariate balance (Love) plot. Standardized mean differences (SMDs) are shown which represent the between‐cohort balance of key covariates cohorts before and after propensity score matching. The vertical dashed line at SMD=0.1 represents the threshold for optimal covariate balance [13,14]. Triangles indicate SMD values before matching, while squares show SMDs after matching. This plot shows improvement in covariate balance following matching, with all covariates having optimal balance after matching. Plot created by Robert J. Trager using R and R studio (version 4.2.2, Vienna, AT [15]) and the ggplot2 package [16]. **Figure S4:** Follow‐up data. A: This graph shows the percentage of patients remaining at each timepoint during follow‐up per cohort, showing the spinal manipulative therapy (SMT) cohort in orange and ibuprofen cohort in blue. This plot uses locally estimated scatterplot smoothing. B: This plot shows the percentage of patients per cohort who had at least the maximum follow‐up time available (i.e., ibuprofen: 86.6%; SMT: 81.1%). Plots were created by Robert J. Trager using R and R studio (version 4.2.2, Vienna, AT [15]) and the ggplot2 package [16]. **Table S1:** Exclusion criteria for all patients. Table S2: Variables controlled for in propensity score matching. **Table S3:** Codes applied to identify opioid use disorder Table S4: Negative control outcomes unrelated to chiropractic spinal manipulation.

## Data Availability

The data that support the findings of this study are openly available in figshare at https://doi.org/10.6084/m9.figshare.28431131. Minimal, deidentified, aggregated datasets used for the baseline characteristics, our primary outcome, and propensity score density and cumulative incidences are available in a public figshare repository (https://doi.org/10.6084/m9.figshare.28431131).
