# Supplementary material for: Association Between Spinal Manipulative Therapy for Low Back Pain With or Without Sciatica and Opioid Use Disorder: A Retrospective Cohort Study
Source: Health Sci Rep. 2025 Sep 19;8(9):e71267. doi: 10.1002/hsr2.71267 (PMC12447351; doi:10.1002/hsr2.71267)
Supplement: Supplementary file 1 — Figure S1: Graphical representation of study design. The vertical gray arrow indicates the index date (cohort entry date ‐‐ day 0), representing is the when spinal manipulative therapy (SMT) or ibuprofen are received and other criteria are met. Text and boxes describe study eligibility criteria which were assessed during time windows ([#, #]) in days relative to the index date. Figure created by Robert J. Trager using a Creative Commons template from Wang et al [1]. Figure S2: Propensity score density graph. Density scores before (A) and after (B) matching. Orange bars represent the chiropractic spinal manipulative therapy (SMT) cohort while blue bars represent the ibuprofen control cohort. After matching, densities overlap closely (shown in gray), suggesting adequate balance of covariates. Figure S3: Covariate balance (Love) plot. Standardized mean differences (SMDs) are shown which represent the between‐cohort balance of key covariates cohorts before and after propensity score matching. The vertical dashed line at SMD=0.1 represents the threshold for optimal covariate balance [13,14]. Triangles indicate SMD values before matching, while squares show SMDs after matching. This plot shows improvement in covariate balance following matching, with all covariates having optimal balance after matching. Plot created by Robert J. Trager using R and R studio (version 4.2.2, Vienna, AT [15]) and the ggplot2 package [16]. Figure S4: Follow‐up data. A: This graph shows the percentage of patients remaining at each timepoint during follow‐up per cohort, showing the spinal manipulative therapy (SMT) cohort in orange and ibuprofen cohort in blue. This plot uses locally estimated scatterplot smoothing. B: This plot shows the percentage of patients per cohort who had at least the maximum follow‐up time available (i.e., ibuprofen: 86.6%; SMT: 81.1%). Plots were created by Robert J. Trager using R and R studio (version 4.2.2, Vienna, AT [15]) and the ggplot2 package [16]. Table S1: Ex [file HSR2-8-e71267-s001.pdf]

Supplemental File 1

For: Association between spinal manipulative therapy for low back pain with or without sciatica and opioid use disorder: a retrospective cohort study

Robert J. Trager, Zachary A. Cupler, Jordan A. Gliedt, Ryan Fischer, Roshini Srinivasan, Hannah Thorfinnson

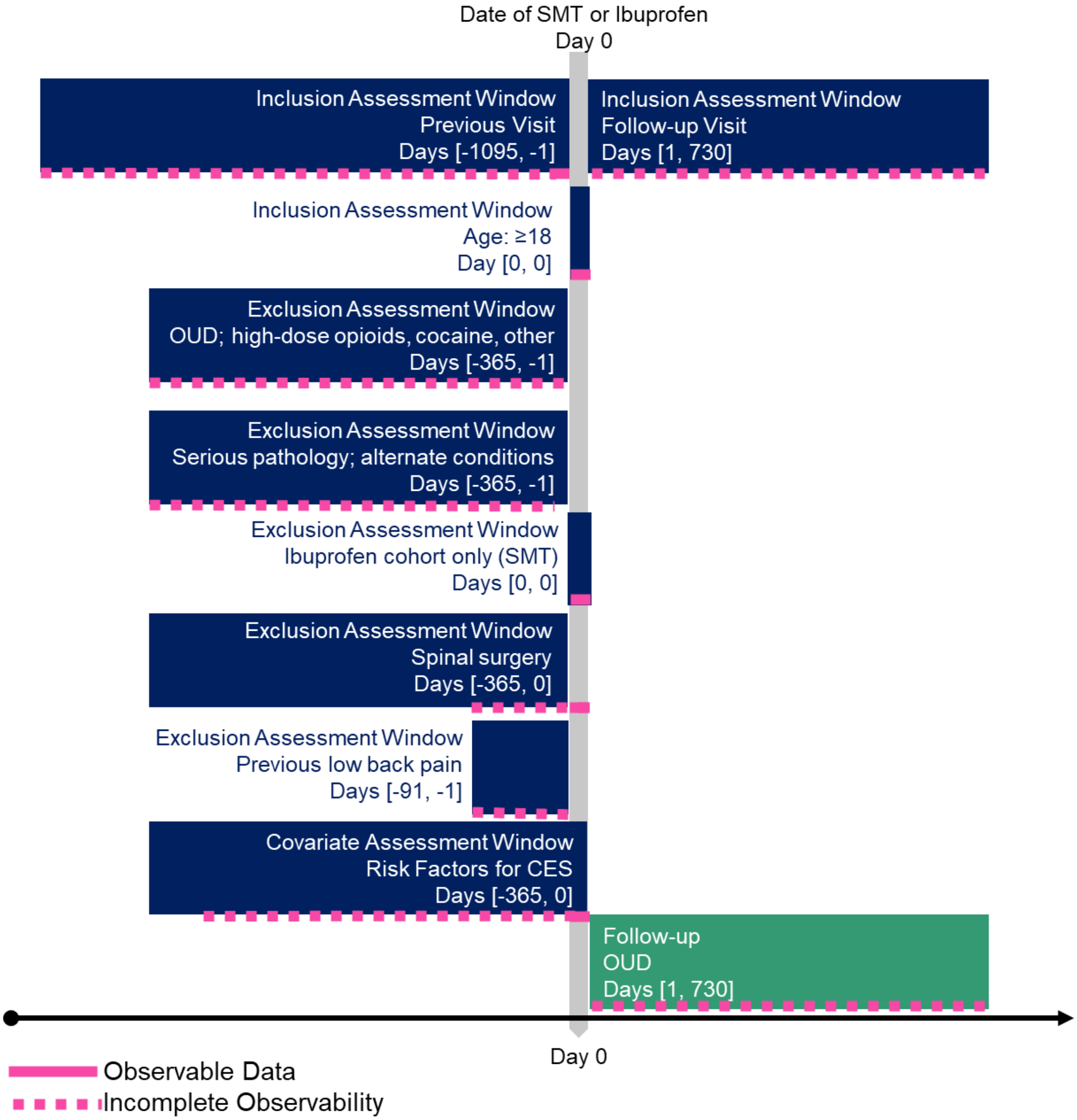

Figure S1: Graphical representation of study design. The vertical gray arrow indicates the index date (cohort entry date -- day 0), representing is the when spinal manipulative therapy (SMT) or ibuprofen are received and other criteria are met. Text and boxes describe study eligibility criteria which were assessed during time windows ([#, #]) in days relative to the index date. Figure created by Robert J. Trager using a Creative Commons template from Wang et al [1].

Table S1: Exclusion criteria for all patients. Abbreviations: Central nervous system (CNS); Current Procedural Terminology (CPT); International Classification of Diseases, 10th Revision (ICD-10); Logical Observation Identifiers Names and Codes (LOINC®), normalized names for clinical drugs (RxNorm); custom TriNetX code (NA)

**Code**	**Description**	**Window (days)**
Serious pathology (ICD-10)		
C00-C96	Malignant neoplasm	-365 to 0
G06	Intracranial and intraspinal abscess and granuloma	-365 to 0
G83.4	Cauda equina syndrome	-365 to 0
M46	Other inflammatory spondyloarthropathies (includes infections)	-365 to 0
M48.4	Fatigue fracture of vertebra	-365 to 0
M48.5	Collapsed vertebra, not elsewhere classified	-365 to 0
Z51.5	Encounter for palliative care	-365 to 0
Substance Use Disorders (ICD-10)		
F11	Opioid related disorders (includes heroin)	-365 to 0
F15	Other stimulant related disorders (includes methamphetamines)	-365 to 0
F14	Cocaine related disorders	-365 to 0
F15	Other stimulant-related disorders (includes amphetamines)	-365 to 0
Alternate conditions (ICD-10)		
G35	Multiple sclerosis	-365 to 0
G54.1	Lumbosacral plexus disorders	-365 to 0
G95	Other and unspecified diseases of spinal cord	-365 to 0
G99.2	Myelopathy in diseases classified elsewhere	-365 to 0
M41	Scoliosis	-365 to 0
M43.16	Spondylolisthesis, lumbar region	-365 to 0
M43.17	Spondylolisthesis, lumbosacral region	-365 to 0
M48.0	Spinal stenosis (includes lumbar stenosis with neurogenic claudication)	-365 to 0
M50.0	Cervical disc disorder with myelopathy	-365 to 0
M51.0	Thoracic, thoracolumbar, and lumbosacral intervertebral disc disorders with myelopathy	-365 to 0
M96.1	Postlaminectomy syndrome, not elsewhere classified	-365 to 0
O00-O9A	Pregnancy	-280 to 365
Z98.1	Arthrodesis status	-365 to 0
Medications		
7242	Naloxone	-365 to 0
CN101 (VA)	Opioid analgesics	-365 to 0
N02A (ATC)	Opioids	-365 to 0
Z79.891 (ICD-10)	Long term (current) use of opiate analgesic	-365 to 0
Tests		
3397-7 (LOINC)	Cocaine in urine (presence)	-365 to 0
3414-0 (LOINC)	Buprenorphine in urine (presence)	-365 to 0
19550-3 (LOINC)	Methadone in urine (presence)	-365 to 0
19554-5 (LOINC)	Methamphetamine in urine (presence)	-365 to 0
59673-4 (LOINC)	Fentanyl in urine (presence)	-365 to 0
LG7013-8 (TNX)	Opiates in urine (presence)	-365 to 0
Post- surgery		
1004038 (CPT)	Surgical procedures on the spine (vertebral column)	-365 to 0
1002796 (CPT)	Anesthesia	-1 to 0
27130 (CPT)	Arthroplasty, acetabular and proximal femoral prosthetic replacement (total hip arthroplasty), with or without autograft or allograft	-1 to 0
New care episode for low back pain with or without leg pain		
Various	Lumbosacral root disorders (G54.4), radiculopathy, lumbar, lumbosacral, sacral, or coccygeal region (M54.16, M54.17, M54.18), sciatica (M54.3), lumbago with sciatica (M54.4), low back pain (M54.5)	-91 to -1
Opioid use disorder (see Table S3)		-365 to 0

Table S2: Variables controlled for in propensity score matching. Abbreviations: Anatomical Therapeutic Chemical Classification (ATC), Current Procedural Terminology (CPT), normalized names for clinical drugs (RxNorm); International Classification of Diseases, 10th Revision (ICD-10); Veterans Health Administration National Drug File (VA)

**Variable/Code**	**Description**	**Reference(s)**
Demographics	Patient age, sex, race, and ethnicity	[2,3]
Diagnoses (ICD-10)		
B18.2	Chronic viral hepatitis C	[2]
E55	Vitamin D deficiency	[4]
F10-F19	Mental and behavioral disorders due to psychoactive substance use, including alcohol, opioid, and other substances	[2,3]
F10	Alcohol related disorders	[2,3]
F17	Nicotine dependence (includes smoking, vaping)	[2,3]
F30-F39	Mood disorders (includes depression, bipolar)	[2]
F40-F48	Anxiety, dissociative, stress-related, somatoform and other nonpsychotic mental disorders	[2]
G54	Nerve root and plexus disorders	[2]
G89.2	Chronic pain, not elsewhere classified	[5]
M05-M14	Inflammatory polyarthropathies	[2]
M19	Other and unspecified osteoarthritis	[2]
M48.02	Lumbar stenosis with neurogenic claudication	[2]
M54.1	Radiculopathy	[2]
M54.3	Sciatica	[2]
M54.4	Lumbago with sciatica	[2]
R45.851	Suicidal ideations	[3]
T14.91	Suicide attempt	[3]
Z55-Z65	Adverse socioeconomic and psychosocial circumstances	[3]
Z72.0	Tobacco use	[2,3]
Z81.4	Family history of other psychoactive substance abuse and dependence	[6]
Z91.4	Personal history of psychological trauma, not elsewhere classified	[2]
Z91.5	Personal history of self-harm	[3]
Visits and prescription medications		
1013729 (CPT)	Critical Care Services	[5]
1003143 (CPT)	Surgery	[5]
AD100 (VA)	Alcohol deterrents	[2,3]
CN300 (VA)	Sedatives/hypnotics (includes benzodiazepines)	[2,3]
CN800 (VA)	Central nervous system stimulants	[3]
VA000 (VANDF)	Medications (any)	NA

Table S3: Codes applied to identify opioid use disorder

Code(s)	Definition	Reference
F11 (ICD-10)	Opioid related disorders	[7–9]
T40.0, T40.2, T40.3, T40.4 (ICD-10)	Poisoning by, adverse effect of and underdosing of opium, other opioids, methadone, and other synthetic narcotics	[7,8,10]
J2310, J2315 (HCPCS)	Injection, naloxone	[8,10]
J0570, J0571, J0572, J0573, J0574, J0575, J0592 (HCPCS)	Buprenorphine and/or naloxone administration	[7–10]
S0109 (HCPCS)	Methadone, oral, 5 mg	[7,10]
J1230 (HCPCS)	Injection, methadone hcl, up to 10 mg	[7,8]
1819 (RxNorm)	Buprenorphine	[8,11]
6813 (RxNorm)	Methadone	[8,11]
Q9991, Q9992 (HCPCS)	Injection, buprenorphine extended-release (sublocade)	[9]
HZ81ZZZ (ICD-10-PCS)	Medication management for substance abuse treatment, methadone maintenance	[8]
HZ91ZZZ (ICD-10-PCS)	Pharmacotherapy for substance abuse treatment, methadone maintenance	[8]
HZ85ZZZ (ICD-10-PCS)	Medication management for substance abuse treatment, naloxone	[8]
HZ95ZZZ (ICD-10-PCS)	Pharmacotherapy for substance abuse treatment, naloxone	[8]
H0020 (HCPCS)	Alcohol and/or drug services; methadone administration and/or service (provision of the drug by a licensed program)	[10]
G2067 through G2080 (HCPCS)	Provision of the services by a Medicare-enrolled opioid treatment program	[9,12]

Table S4: Negative control outcomes unrelated to chiropractic spinal manipulation. Abbreviations: Current Procedural Terminology (CPT); International Classification of Diseases, 10th Revision (ICD-10)

Variable	Definition
1022231 (CPT)	Colonoscopy
18631 (CPT)	Azithromycin
J00-J06 (ICD-10)	Acute upper respiratory infections
K35 (ICD-10)	Acute appendicitis

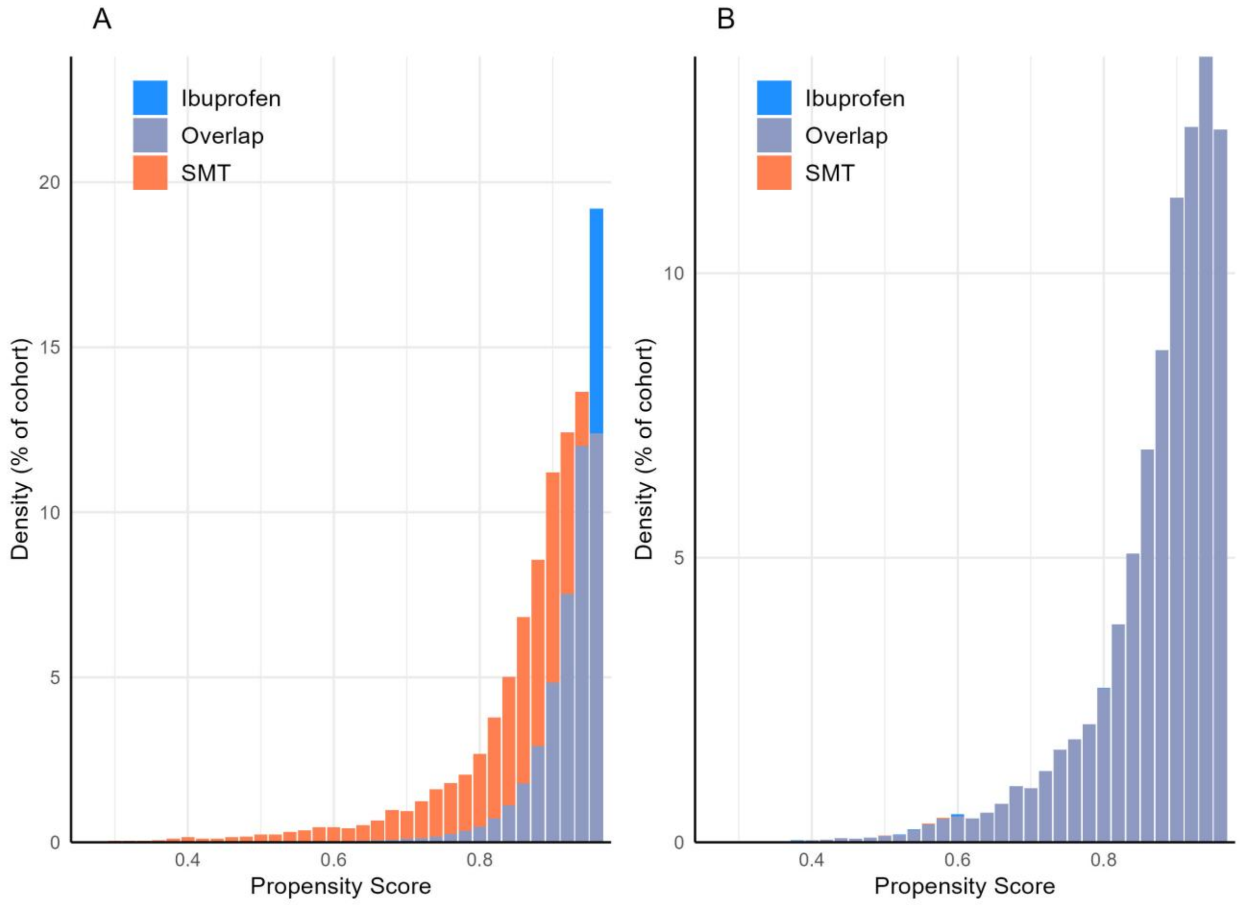

Figure S2: Propensity score density graph. Density scores before (A) and after (B) matching. Orange bars represent the chiropractic spinal manipulative therapy (SMT) cohort while blue bars represent the ibuprofen control cohort. After matching, densities overlap closely (shown in gray), suggesting adequate balance of covariates.

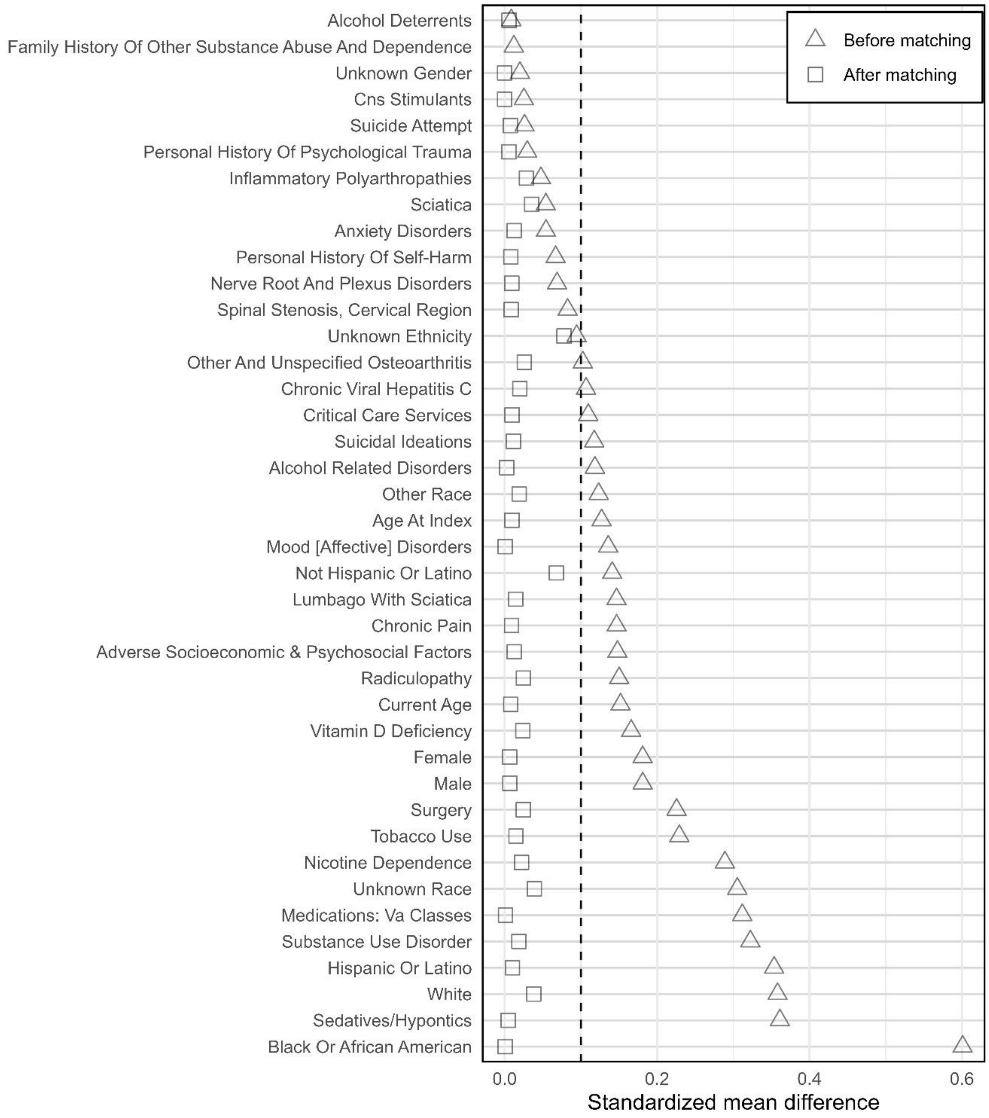

Figure S3: Covariate balance (Love) plot. Standardized mean differences (SMDs) are shown which represent the between-cohort balance of key covariates cohorts before and after propensity score matching. The vertical dashed line at SMD=0.1 represents the threshold for optimal covariate balance [13,14]. Triangles indicate SMD values before matching, while squares show SMDs after matching. This plot shows improvement in covariate balance following matching, with all covariates having optimal balance after matching. Plot created by Robert J. Trager using R and R studio (version 4.2.2, Vienna, AT [15]) and the ggplot2 package [16].

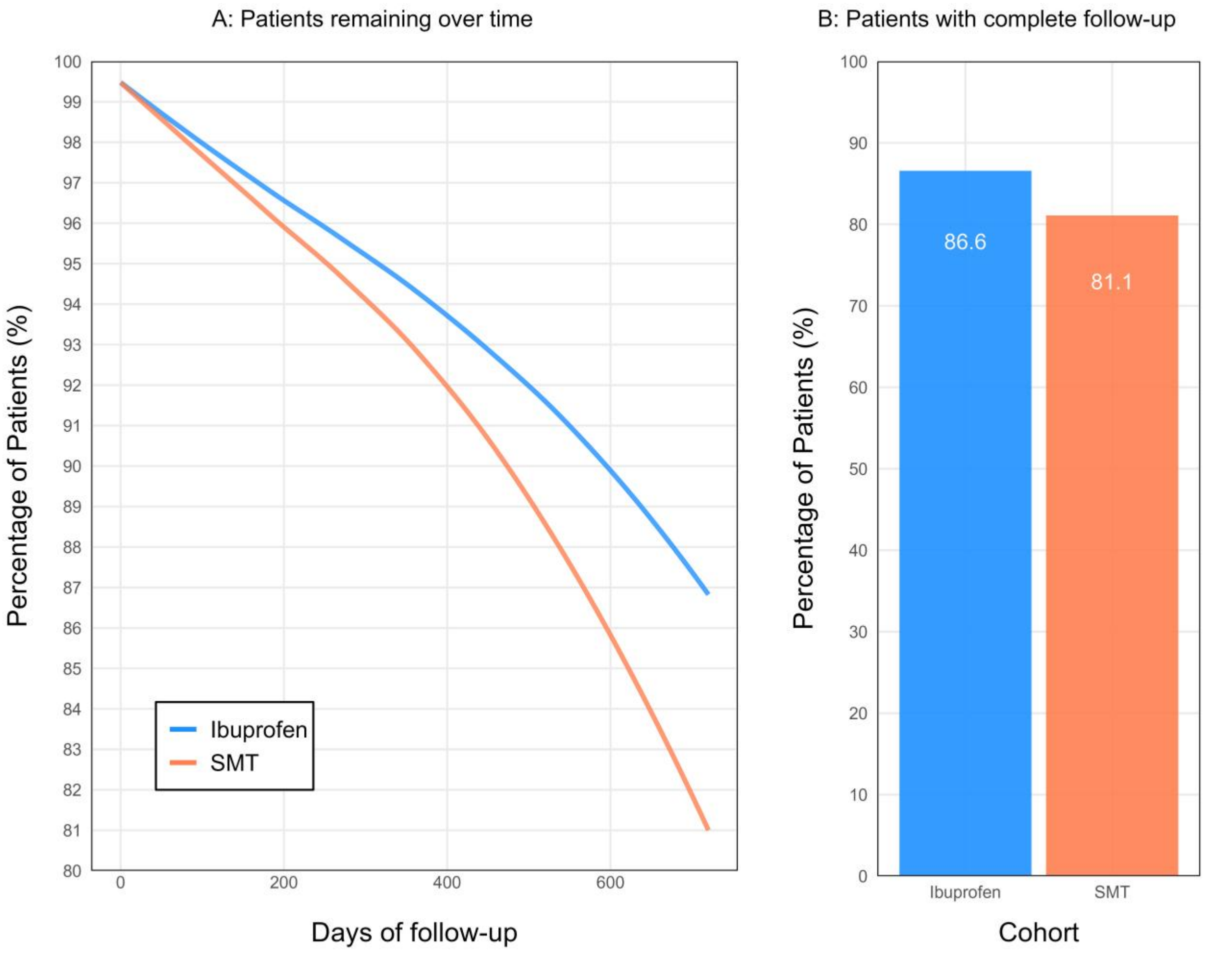

Figure S4. Follow-up data. A: This graph shows the percentage of patients remaining at each timepoint during follow-up per cohort, showing the spinal manipulative therapy (SMT) cohort in orange and ibuprofen cohort in blue. This plot uses locally estimated scatterplot smoothing. B: This plot shows the percentage of patients per cohort who had at least the maximum follow-up time available (i.e., ibuprofen: 86.6%; SMT: 81.1%). Plots were created by Robert J. Trager using R and R studio (version 4.2.2, Vienna, AT [15]) and the ggplot2 package [16].

References

1. Wang SV, Schneeweiss S. A Framework for Visualizing Study Designs and Data Observability in Electronic Health Record Data. Clin Epidemiol. 2022 Apr 29;14:601–8.

2. Cragg A, Hau JP, Woo SA, Kitchen SA, Liu C, Doyle-Waters MM, Hohl CM. Risk Factors for Misuse of Prescribed Opioids: A Systematic Review and Meta-Analysis. Ann Emerg Med. 2019 Nov 1;74(5):634–46.

3. Montiel Ishino FA, McNab PR, Gilreath T, Salmeron B, Williams F. A comprehensive multivariate model of biopsychosocial factors associated with opioid misuse and use disorder in a 2017–2018 United States national survey. BMC Public Health. 2020 Nov 18;20(1):1740.

4. Bryant BM, Eaton E, Li L. A Systematic Review of Opioid Use Disorder and Related Biomarkers. Front Psychiatry [Internet]. 2021 Aug 11 [cited 2024 Aug 13];12. Available from: https://www.frontiersin.org/journals/psychiatry/articles/10.3389/fpsyt.2021.708283/full

5. Baumann L, Bello C, Georg FM, Urman RD, Luedi MM, Andereggen L. Acute Pain and Development of Opioid Use Disorder: Patient Risk Factors. Curr Pain Headache Rep. 2023 Sep 1;27(9):437–44.

6. Schepis TS, Wastila L, McCabe SE. Family History of Substance Use Disorder and Likelihood of Prescription Drug Misuse in Adults 50 and Older. Aging Ment Health. 2023 May;27(5):1020–7.

7. Use of Medication-Assisted Treatment for Opioid Use Disorders in Employer-Sponsored Health Insurance: Final Report [Internet]. ASPE. 2019 [cited 2024 Aug 14]. Available from: https://aspe.hhs.gov/reports/use-medication-assisted-treatment-opioid-use-disorders-employer-sponsored-health-insurance-final-0

8. White DG, Adams NB, Brown AM, O'Jiaku-Okorie A, Badwe R, Shaikh S, Adegboye A. Enhancing Identification of Opioid-involved Health Outcomes Using National Hospital Care Survey Data. Vital Health Stat Ser 1 Programs Collect Proced. 2021 Oct;(188):1–31.

9. Nakamoto CH, Huskamp HA, Donohue JM, Barnett ML, Gordon AJ, Mehrotra A. Medicare Payment for Opioid Treatment Programs. JAMA Health Forum. 2024 Jul 19;5(7):e241907.

10. Busch AB, Greenfield SF, Reif S, Normand SLT, Huskamp HA. Outpatient care for opioid use disorder among the commercially insured: Use of medication and psychosocial treatment. J Subst Abuse Treat. 2020 Aug;115:108040.

11. Segal Z, Radinsky K, Elad G, Marom G, Beladev M, Lewis M, Ehrenberg B, Gillis P, Korn L, Koren G. Development of a machine learning algorithm for early detection of opioid use disorder. Pharmacol Res Perspect. 2020 Nov 16;8(6):e00669.

12. Opioid Treatment Programs (OTPs) Medicare Billing and Payment Fact Sheet. Centers for Medicare & Medicaid Services; 2020.

13. Austin PC. Balance diagnostics for comparing the distribution of baseline covariates between treatment groups in propensity-score matched samples. Stat Med. 2009;28(25):3083–107.

14. Stuart EA, Lee BK, Leacy FP. Prognostic score–based balance measures can be a useful diagnostic for propensity score methods in comparative effectiveness research. J Clin Epidemiol. 2013 Aug 1;66(8, Supplement):S84-S90.e1.

15. R Core Team. R: A Language and Environment for Statistical Computing [Internet]. Vienna, Austria: R Foundation for Statistical Computing; 2022. Available from: https://www.R-project.org/

16. Wickham H. ggplot2: Elegant Graphics for Data Analysis [Internet]. Springer-Verlag New York; 2016. Available from: https://ggplot2.tidyverse.org
